# Improving protein-ligand binding site prediction accuracy by classification of inner pocket points using local features

**DOI:** 10.1186/s13321-015-0059-5

**Published:** 2015-04-01

**Authors:** Radoslav Krivák, David Hoksza

**Affiliations:** Department of Software Engineering, Charles University in Prague, Prague, Czech Republic

**Keywords:** Ligand binding site, Protein pocket, Binding site prediction, Pocket score, Molecular recognition, Machine learning, Random forests

## Abstract

**Background:**

Protein-ligand binding site prediction from a 3D protein structure plays a pivotal role in rational drug design and can be helpful in drug side-effects prediction or elucidation of protein function. Embedded within the binding site detection problem is the problem of pocket ranking – how to score and sort candidate pockets so that the best scored predictions correspond to true ligand binding sites. Although there exist multiple pocket detection algorithms, they mostly employ a fairly simple ranking function leading to sub-optimal prediction results.

**Results:**

We have developed a new pocket scoring approach (named PRANK) that prioritizes putative pockets according to their probability to bind a ligand. The method first carefully selects pocket points and labels them by physico-chemical characteristics of their local neighborhood. Random Forests classifier is subsequently applied to assign a ligandability score to each of the selected pocket point. The ligandability scores are finally merged into the resulting pocket score to be used for prioritization of the putative pockets. With the used of multiple datasets the experimental results demonstrate that the application of our method as a post-processing step greatly increases the quality of the prediction of Fpocket and ConCavity, two state of the art protein-ligand binding site prediction algorithms.

**Conclusions:**

The positive experimental results show that our method can be used to improve the success rate, validity and applicability of existing protein-ligand binding site prediction tools. The method was implemented as a stand-alone program that currently contains support for Fpocket and Concavity out of the box, but is easily extendible to support other tools. PRANK is made freely available at http://siret.ms.mff.cuni.cz/prank.

**Electronic supplementary material:**

The online version of this article (doi:10.1186/s13321-015-0059-5) contains supplementary material, which is available to authorized users.

## Background

Accurate prediction of ligand-binding sites, often simply called pockets, from a 3D protein structure plays a pivotal role in rational drug design [1,2] and can be helpful in drug side-effects prediction [3] and elucidation of protein function [4]. Ligand-binding sites are usually found in deep protein surface cavities, but it should be emphasized that not all binding sites are found in deep cavities. Although empirical studies show that the actual ligand-binding sites tend to coincide with the largest and deepest pocket on the protein’s surface [5,6], there exist cases where ligands are found binding to rather exposed shallow clefts [7,8].

Plethora of pocket detection methods, that employ variety of different strategies, are currently available. These include purely geometric methods, energetic methods and methods that make use of evolutionary conservation (see below). All these methods take a protein structure as an input and produce an ordered list of putative pockets, which represent the locations on the protein surface where ligands are expected to bind. Not all reported pockets usually correspond to true binding sites, but it is expected that entries at the top of the ordered list correspond to regions with the highest probability of being a true binding site. Although it is not unusual for one protein to have more than one ligand-binding site, the number of putative pockets predicted by pocket detection methods tends to be much higher than the number of actual known positives. The accuracy of a pocket prediction method is then evaluated by its ability to yield the true (experimentally confirmed) binding sites among the top-*n* putative pockets on its output (where *n* is usually taken to be 1, 3 or 5).

As the list of predicted pockets contains false positives, ordering of the pockets, i.e. pocket ranking, plays an important role and substantially contributes to the overall accuracy of the prediction method. More importantly, correct pocket ranking is of practical utility: it helps to prioritize subsequent efforts concerned with the predicted pockets, such as molecular docking or virtual screening.

While many ligand-binding site detection approaches employ complex and inventive algorithms to locate the pockets, the final ranking is often done by a simple method such as ordering by size or scoring pockets by a linear combination of few pocket descriptors. In the present study we are introducing a novel pocket ranking algorithm based on machine learning that can be used as a post-processing step after the application of a pocket prediction method and thus improve its accuracy. We demonstrate that applying this re-ordering step substantially improves identification success rates of two pocket prediction methods, Fpocket [9] and ConCavity [10], on several previously introduced datasets.

### Pocket detection approaches

In the last few years, we have been able to observe increased interest in the field of pocket detection indicated by a number of recently published reviews [2,11,12], as well as by the influx of new detection methods. The pocket detection algorithms can be categorized based on the main strategy they adopt in the process of binding site identification. Those strategies and their representative methods shall be briefly reviewed in the following paragraphs.

#### Geometry based methods

The geometrical methods focus mainly on the algorithmic side of the problem of finding concave pockets and clefts on the surface of a 3D structure. Some methods are purely geometrical (LIGSITE [13], LIGSITE^cs^ [14], PocketPicker [5]), while others make use of additional physico-chemical information like polarity or charge (MOE SiteFinder [15], Fpocket [9]).

#### Energy based methods

The energy based methods build on the approximation of binding potentials or binding energies [16]. They place various probes on the grid points around the protein’s surface and calculate interaction energies of those points with the use of underlying force field software. That results in higher computational demands of these methods [17]. Representative examples of the energy based methods include Q-SiteFinder [18], SiteHound [8], dPredGB [19] or the method by Morita et al. [20].

#### Evolutionary and threading based methods

The sequence-based evolutionary conservation approaches are based on the presumption that functionally important residues are preferentially conserved during the evolution because natural selection acts on function [21]. In LIGSITE^csc^ [14], a sequence conservation measure of neighboring residues was used to re-rank top-3 putative pockets calculated by LIGSITE^cs^, which lead to an improved success rate (considering top-1 pocket). In ConCavity [10], unlike in LIGSITE^csc^, the sequence conservation information is used not only to re-rank pockets, but it is also integrated directly into the pocket detection procedure. An example of an evolutionary based method which takes into account the structural information is FINDSITE [22,23]. It is based on the observation that even distantly homologous proteins usually have similar folds and bind ligands at similar locations. Thus at first ligand-bound structural templates are selected from the database of already known protein-ligand complexes by a threading (fold recognition) algorithm. The used threading algorithm is not based only on sequence similarity, but it also combines various scoring functions designed to match structurally related target/template pairs [24]. Found homologous structures are subsequently aligned with the target protein by a global structural alignment algorithm. Positions of ligands on superimposed template structures are then clustered into consensus binding sites.

#### Consensus methods

The consensus methods are essentially meta approaches combining results of other methods. The prominent example is MetaPocket [25]. The recently introduced updated version, MetaPocket 2.0 [26], aggregates predicted sites of 8 different algorithms (among them the aforementioned LIGSITE^cs^, Q-SiteFinder, Fpocket and ConCavity) by taking top 3 sites from each method. The authors demonstrated that MetaPocket performed better than any of the individual methods alone.

### Ranking algorithms

Given that every pocket identification algorithm is basically a heuristic it needs to incorporate a scoring function providing a measure of confidence in given prediction. A simple strategy for scoring putative pockets, one that is probably most commonly used, is ordering pockets by a single descriptor — like size (volume), pocket depth, surface area or the overall hydrophobicity. Another strategy for scoring pockets is to combine several pocket descriptors. Fpocket, for example, uses a linear combination of 5 such descriptors which parameters were optimized on a training dataset. The same approach was also successfully applied in recent druggability prediction methods [27,28]. In ConCavity, the ranking procedure considers overall pocket evolutionary conservation score that is projected onto pocket grid probes. One study that focused solely on ranking of pockets previously found by other pocket detection algorithms introduced an approach based on amino acid composition and relative ligand binding propensities of different amino acids termed PLB index [29] (we compare our proposed method with PLB index in results section).

It has been suggested that pocket identification and pocket ranking are independent tasks and therefore should be evaluated separately [30].

It seems that pocket detection methods that have achieved the highest success rates in the aforementioned benchmark are those with more sophisticated ranking algorithms. It has also been suggested that the total coverage (i.e. identification success rate considering all predicted pockets without regard to the ordering) of many algorithms is actually close to 100% [30]. While our experiments do not support such a strong claim they, nevertheless, show that there is indeed a big difference between success rate with regards to top 1, top 3 binding sites and the total coverage. Therefore, there is room for improvement by introducing a more precise and sophisticated ranking algorithm that would rank the identified true pockets higher than the false ones.

#### Performance of existing methods

Considering that the goal of our method is to increase the performance of the existing state of the art methods we have to raise a question regarding their actual performance. It has been acknowledged that the field of ligand-binding site prediction lacks standardized and widely accepted benchmarking datasets and guidelines [30,31]. In the studies introducing the individual methods, their performance was usually compared to a couple of existing methods with (somewhat expectedly) favorable results, reporting success rates around 90% regarding the top 3 and 70% considering the top 1 predicted sites. The latest review [31] represents the first independent attempt to systematically assess the performance of the pocket detection methods, although only a limited set of 8 representative methods has been considered. It has challenged the previously reported high success rates of the pocket prediction programs. With the exception of FINDSITE, identification success rates of all methods on the new dataset were considerably lower than previously reported (closer to 50% rather than the often reported 70% for top 1 prediction). FINDSITE achieved clearly the best results, but only with the help of a comprehensive threading library that contained proteins highly similar to those from the benchmarking dataset. It was demonstrated that when those were removed from the library, success rates of FINDSITE dropped to the level of other methods [31].

## Methods

We are introducing here a new pocket ranking method PRANK that can be used to increase the performance of existing pocket prediction methods. Thus the input of the method is a list of predicted putative pockets and its goal is to prioritize the list in such a way that the true pockets appear at the top of that list. PRANK is a machine learning method which is based on predicting ligandability of specific pocket points near the pocket surface. These points represent possible locations of contact atoms of a putative ligand. By aggregating predictions of those points PRANK outputs a score to be used for the re-ranking of the putative pockets. Thus, unlike previous studies that applied machine learning in the context of protein binding site prediction [32-37], we focused on the classification of inner pocket points rather than the classification of exposed amino acid residues or whole pockets. The following list outlines the PRANK method (see also Figure 1):

1. Sampling inner pocket points from Connolly surface of the protein.

**Figure 1:**
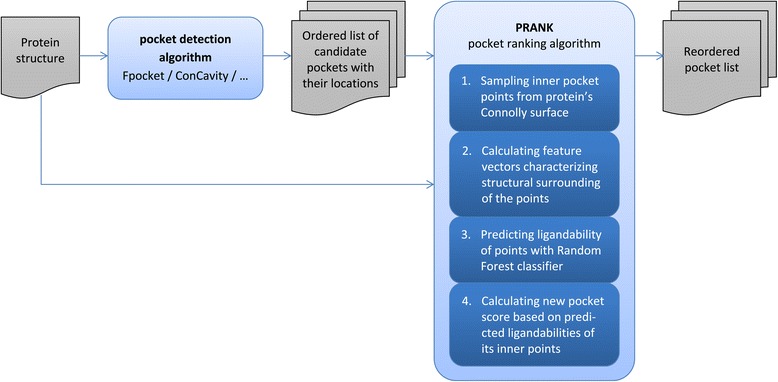
Flowchart of the PRANK pocket ranking approach.
2. Calculating feature descriptors of the sampled points based on their local chemical neighborhood.
  Computing property vectors of chosen protein’s solvent exposed atoms.
  Projecting distance weighted properties of the adjacent protein atoms onto the sampled inner pocket points.
  Computing additional inner pocket points specific features.
3. Predicting ligandability of the sampled inner pocket points by random forests classifier using their feature vectors.
4. Aggregating predictions into the final pocket score.

**Figure 2:**
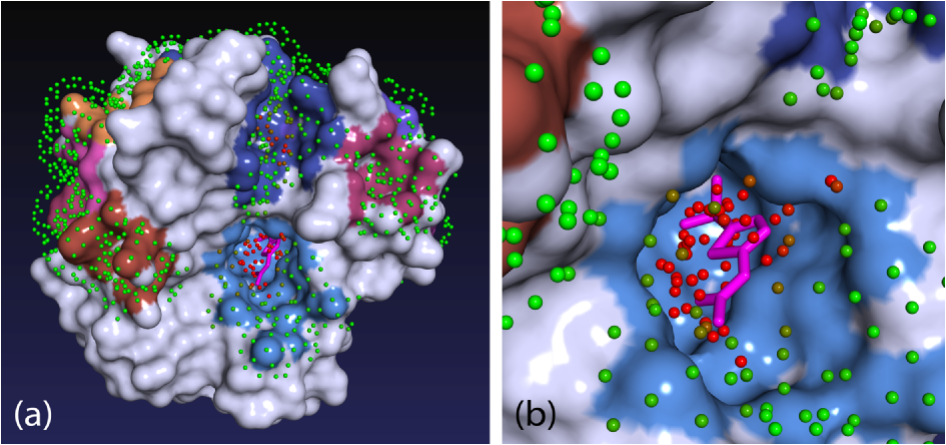
Visualization of inner pocket points.**(a)** Displayed is protein 1AZM from DT198 dataset bound to one ligand (magenta). Fpocket predicted 13 pockets that are depicted as colored areas on the protein surface. To rank these pockets, the protein was first covered with evenly spaced Connolly surface points (probe radius 1.6 Å) and only the points adjacent to one of the pockets were retained. Color of the points reflects their ligandability (green = 0…red = 0.7) predicted by Random Forest classifier. PRANK algorithm rescores pockets according to the cumulative ligandability of their corresponding points. Note that there are two clusters of ligandable points in the picture, one located in the upper dark-blue pocket and the other in the light-blue pocket in the middle. The light-blue pocket, which is in fact the true binding site, contains more ligandable points and therefore will be ranked higher. **(b)** Detailed view of the binding site with ligand and inner pocket points.

Individual steps are described in greater detail in following sections. For the visualization of classified pocket points see Figure 2.

### Pocket representation

To represent a pocket, PRANK first computes a set of its *inner points* by selecting evenly spaced points lying on the Connolly surface [38] that lie in the distance of at most 4 Å from the closest heavy pocket atom. This method of choosing points to represent a pocket is similar to the one used by Morita et al. [20], although we deliberately use only one Connolly surface layer with optimized probe radius of 1.6 Å. Thus PRANK utilizes only points in a relatively short belt around the pocket surface as the bonding between ligand and protein takes place in this area.

Next, PRANK assigns a feature vector to each of the inner points. The feature vector is built in two steps: first, it calculates feature vectors for specific pocket atoms (*AFVs*) which are then aggregated into feature vectors of the inner points (*IFVs*).

The AFVs are computed only for pocket atoms located in the atomic neighborhood of any inner point. The atomic neighborhood of point *P* is defined as:

(1) $$  \begin{aligned}  \mathrm{A}(\textit{P}) =&\; \left\{\textrm{heavy solvent exposed protein atoms within 8}\right.\\ &\left.\text{{\AA} radius around} \,\textit{P} \right\} \end{aligned}  $$

The features forming the AFVs include two types of features: residue level features and atomic level features. The residue level features are characteristics of residues inherited by their constituent atoms. Such features include, e.g., physico-chemical properties of standard amino acids or hydropathy index of amino acids [39]. The atomic levels features are specific to individual atoms meaning that different atoms within one amino acid can have different values of those features. Examples of such features are physico-chemical properties of individual amino acid atoms adopted from VolSite druggability prediction study [40] or statistical ligand-binding propensities of amino acid atoms [41] (see Additional file 1: Listings for the complete feature list).

To calculate the feature vector of an inner pocket point (IFV), the AFVs from its atomic neighborhood are aggregated using a simple aggregation function and concatenated with a vector of features computed specifically for that point from its local neighborhood. These inner point features include the number of H-bond donors and acceptors, B-factor of structure atoms or protrusion index [42] The following aggregation function is used to project the pocket atoms feature vectors onto the inner points:

(2) $$   \textrm{IFV}(P) = \sum_{\mathrm{A}_{i}\in\ \mathrm{A}(P)} \textrm{AFV}\left(\mathrm{A}_{i}\right) \cdot w(\text{dist}(P,\mathrm{A}_{i})) \quad || \quad \text{FV}(P),  $$

where FV is the vector of the inner points specific features and *w* is a distance weight function :

(3) $$  w(d) = 1 - d/8.  $$

We evaluated several types of weight functions with different parameters (among them quadratic, Gaussian and sigmoid), but in the end we selected the present simple linear function which had produced the best results in the cross-validation experiments.

It also needs to be emphasized that all of the features included in the vectors are local, which means that they are calculated only based on the immediate spatial neighborhood of the points. No regard is taken to the shape and properties of the whole pocket or protein. Although the 8 Å cutoff radius by which we define chemical neighborhood can encompass considerable part of the whole pocket, immediate surrounding atoms have more influence thanks to the fact that we weight their contribution by distance (see Equation 3). Inner pocket points from different parts of the pocket can therefore have very different feature vectors. We propose that this locality has some positive impact on the generalization ability of the model.

One possible negative implication of considering only local features could be that local features are not sufficient to account for ligand binding quality of certain regions of protein surface since some ligand positions could be fixed by few relatively distant non-covalent bonds. However, our results show that in spite of that concern our local approach leads to practical improvements.

### Classification-based ligandability prediction

Similarly to other studies that were trying to predict whether exposed residues of a protein are ligand binding or not, we used a machine learning approach to predict the ligandability of inner pocket points. The ligandability prediction is a binary classification problem for supervised learning. Training datasets of inner pocket points were generated as follows. For a given protein dataset with candidate pockets (e.g. CHEN11 dataset with Fpocket predictions) we merged all sampled inner pocket points and labeled as positive those located within 2.5 Å distance to any ligand atom. The resulting point datasets were highly imbalanced in terms of positives and negatives since most of the candidate pockets and their points were not true ligand binding sites (e.g. CHEN11-Fpocket dataset contained 451,104 negative and 30,166 positive points resulting in 15:1 ratio). Compensation techniques such as oversampling, undersampling and cost-sensitive learning are sometimes applied in such scenarios, but in our experiments they only led to notable degradation of the generalization ability of a trained classifier (i.e. performance on other datasets). The size of the point dataset depends on the density of the points sampled from the Connolly surface of a protein. The numerical algorithm that was employed to calculate the Connolly surface [43] is parametrized by an integer tessellation level. Our algorithm uses level 2 by default as higher levels increase the number of points geometrically but do not improve the results.

After preliminary experiments with several machine learning methods we decided to adopt Random Forests [44] as our predictive modelling tool of choice. Random Forests is an ensemble of trees created by using bootstrap samples of training data and random feature selection in tree induction [45]. In comparison with other machine learning approaches, Random Forests are characterized by an outstanding speed (both in learning and execution phase) and generalization ability [44]. Additionally, Random Forests is robust to the presence of a large number of irrelevant variables; it does not require their prior scaling [37] and can cope with complex interaction structures as well as highly correlated variables [46]. The ability of Random Forests to handle correlated variable comes in handy in our case because for example features such as hydrophobicity and hydrophilicity are obviously related.

To report the performance of a classifier, three statistics are commonly reported: precision, recall (also called sensitivity) and Matthews Correlation Coefficient (MCC). MCC is often used to describe the performance of a binary classifier by a single number in scenarios with imbalanced datasets. In such scenarios the predictive accuracy is not an effective assessment index. MCC values range from +1 (perfect prediction), over 0 (random prediction) to −1 (inverse prediction). The performance statistics are calculated as shown below. TP, TN, FP and FP stand for true positive, true negative, false positive, and false negative predictions.

(4) $$  \text{precision} = \frac{\text{TP}}{\text{TP}+\text{FP}}  $$

(5) $$  \text{recall} = \frac{\text{TP}}{\text{TP}+\text{FN}}  $$

(6) $$   \text{MCC} = \frac{\text{TP} \times \text{TN} - \text{FP} \times \text{FN}} {\sqrt{(\text{TP} + \text{FP}) (\text{TP} + \text{FN}) (\text{TN} + \text{FP}) (\text{TN} + \text{FN})} }  $$

### Scoring function

As soon as the classifier is trained it can be used within the PRANK’s scoring function to rescore the putative pockets. To do so we utilize the histogram of class probabilities returned by the random forests classifier for every sampled inner pocket point. Since our problem is binary (a point can either be seen as a pocket point or not) the histogram is an ordered pair [*P*_0_,*P*_1_]. The score is then the sum of predicted squared positive class probabilities of all inner pocket points:

(7) $$  \textrm{PScore} = \sum_{i}^{} (P_{1}(V_{i}))^{2}  $$

Squaring the probabilities puts more emphasis on the points with probability closer to 1. Originally, we experimented with a mean probability based pocket score where *PScore* was divided by the number of inner points. However, we found that the employed cumulative score steadily gives better results. We attribute it to the fact that the size of a correctly predicted pocket can slightly deviate from the true pocket but it still should be recognized as a true pocket. In an oversized predicted pocket that contains in it a true binding site, dividing by the number of points would lead to the decrease of its score.

The higher the *PScore* of a putative pocket, the higher the probability of it being a true pocket. Thus the very last step involves reordering the putative pockets in the decreasing order of their *PScores*.

### Optimization of parameters

Apart from the hyperparameters of the classifier, our method is parameterized by a number of additional parameters that influence various steps of the algorithm, from sampling inner pocket points to calculating and aggregating the features. Since many parameters have an impact on experiment running times and optimizing all parameters at once would be too costly, we optimized default values of those parameters by linear search, and in some cases by grid search (optimizing two parameters at once). Parameters were optimized with regard to the performance on CHEN11 dataset (see the datasets section) considering averaged results of repeated independent runs of 5-fold cross-validation. The optimized parameters included, for example, the probe radius of Connolly’s surface (1.6 Å), ligand distance threshold to denote positive and negative points (2.5 Å) and the choice of the weight function in the inner points feature vector building step.

### Implementation and efficiency

Our software is implemented in languages Groovy and Java with the help of machine learning framework Weka [47] and bioinformatical libraries BioJava [48] and The Chemistry Development Kit (CDK) [49]. Points on the Connolly’s surface are calculated by a fast numerical algorithm [43] implemented in CDK.

Rescoring is implemented in a parallel fashion with configurable number of working threads and therefore can make use of all of the system’s processor cores. In our experience, running times of our rescoring step were generally lower than the running times of the pocket prediction methods themselves, even on a single thread.

## Experimental

### Datasets

To show that application of PRANK is beneficial irrespective of the test set, we investigated its ability to increase the prediction accuracy on several diverse datasets. The following list briefly introduces those datasets.

- CHEN11 – This dataset includes 251 proteins and 476 ligands which were used to benchmark pocket detection methods in a recent comparative review [31]. It was designed with the intention to non-redundantly cover all SCOP families of ligand binding proteins from PDB. It can be considered as “hard” dataset as most methods performed rather poorly on this dataset.
- ASTEX – Astex Diverse set [50] is a collection of 85 proteins that was introduced as a benchmarking dataset for molecular docking methods.
- UB48 – UB48 [14] contains a set of 48 proteins in a bound and unbound state. It has been the most widely used dataset for comparing pocket detection methods. Since it contains mainly small globular proteins with one stereotypical large binding site it can be seen as a rather “easy” dataset.
- DT198 – a dataset of 198 drug-target complexes [26].
- MP210 – a benchmarking dataset of 210 proteins in bound state introduced in the MetaPocket study [25].

For each dataset we generated predictions using two algorithms, Fpocket and ConCavity, which we use as model examples in our re-ranking experiments. Fpocket was used with its default parameters in version 1.0^a^. ConCavity can be run in two modes depending on whether it makes use of sequence conservation information or not. To execute it in the conservation mode it needs to be provided with pre-calculated residue scores. For this we were relying on the pre-computed sequence conservation files available online at the ConCavity website [51]. However, for several proteins from our datasets the conservation files were not available. For these proteins we executed ConCavity with the conservation option turned off. List of affected proteins is provided in Additional file 1: Listings. Except for the conservation switch, ConCavity was run with default parameters.

Table 1 shows statistics of individual datasets together with the average number of pockets predicted per protein by Fpocket and ConCavity. Evidently, Fpocket produces more putative pockets than ConCavity. This number alone, however, is not conclusive since incorrectly identified pockets can be included. However, the table also shows the total coverage (percentage of identified pockets) which is clearly in favor of Fpocket. Higher number of putative pockets and higher coverage makes Fpocket a better target of a re-ranking algorithm.

**Table 1:** **Datasets statistics**

**Dataset**	**Proteins**	**Ligands**	**#L**	**#P**_**FP**_	**#P**_**CC**_	**Cov**_**FP**_ **[%]**	**Cov**_**CC**_ **[%]**	**LS**	**PS**_**FP**_	**PS**_**CC**_
CHEN11	251	476	1.90	12.41	1.75	71.0	52.3	26.9	38.9	51.0
ASTEX	85	143	1.68	21.58	2.25	81.1	65.7	23.2	41.9	56.9
DT198	198	192	0.97	18.57	2.19	80.2	65.6	20.8	41.2	53.7
MP210	210	288	1.37	14.50	1.99	78.8	68.2	22.8	40.0	50.9
B48	48	54	1.13	12.06	1.96	92.6	81.5	21.9	37.8	44.2
U48	48	54	1.13	11.40	1.79	88.9	77.8	21.9	38.0	46.8

Abbreviations: FP Fpocket, CC ConCavity.

#L: average number of ligands for one protein.

#P: average number of predicted pockets for one protein.

Cov: total coverage – success rate considering all predicted pockets (measured by D_CA_ criterion with 4 Å threshold).

LS: average number of heavy atoms in a relevant ligands (ligand size).

PS: average number of protein surface atoms that belong to a predicted pocket (pocket size).

### Evaluation methodology

To evaluate binding site predictions we followed the evaluation methodology introduced in [31]. Unlike previous studies, it uses the ligand-centric not protein-centric approach to calculate success rates. While the ligand-centric approach to evaluation, for a method to be 100% successful on a protein, we want it to identify every pocket on that protein for every relevant ligand in the dataset, the protein-centric approach only requires every protein to have at least one identified binding site. A pocket is considered successfully identified if at least one pocket (of all predicted pockets or from the top of the list) passes a chosen detection criterion (see below).

Furthermore, instead of reporting success rates for Top-1 or Top-3 predicted pockets, we report results for Top-*n* and Top-(*n*+2) cutoffs, where *n* is the number of known ligand-binding sites of the protein that includes evaluated binding site. This adjustment was made to accommodate for proteins with more than one known binding site (CHEN11 dataset, also introduced in [31] contains on average more than 2 binding sites per protein, see Table 1). Specifically, if a protein contains two binding sites, then Top-1 reporting is clearly insufficient in distinguishing methods which returned a correctly identified pocket in the first position of their result set but differ in the second position. For this reason, using the Top-*n* and Top-(*n*+2) cutoffs is more suitable for the ligand-centric evaluation approach.

#### Pocket detection criteria

Since a predicted pocket does not need to match the real pocket exactly, we need a criterion defining when the prediction is correct. When evaluating PRANK we adopted the following two criteria.

- D_CA_ is defined as the minimal distance between the center of the predicted pocket and any atom of the ligand. A binding site is then considered correctly predicted if D_CA_ is not greater than an arbitrary threshold, which is usually 4 *A* ¨. It is the most commonly used detection criterion that has been utilized in virtually all previous studies.
- D_CC_ is defined as the distance between the center of the predicted pocket and the center of the ligand. It was introduced in the Findsite study [22] to compensate for the size of the ligand.

In several studies, criteria based on volume overlap of pocket and ligand were used in addition to the standard criteria. However, since our method does not change the shape of the predicted pockets, inclusion of a volume overlap based criterion would not influence the resulting pocket ordering. Therefore, we did not include any such a criterion into our evaluation.

## Results and discussion

### Results

To demonstrate the PRANK’s ability to increase the quality of prediction of a pocket prediction method (Fpocket and ConCavity) we performed two types of tests. First, we used the CHEN11 dataset for cross-validation experiments and second, we trained our prediction model on the whole CHEN11 dataset and used this model to evaluate our method on the rest of the datasets. The same model is also distributed as the default model in our software package. The reason to train the final model on the CHEN11 dataset is its structural diversity and the fact that it was compiled to include all known ligands for given proteins. The cross-validation results show the viability of our modelling approach on a difficult dataset (CHEN11), and the evaluation of the final model on the remaining datasets attests the generalization ability and applicability of our software out of the box.

The results, including the performance statistics of the classifier, are summarized in Table 2. The *Top-n* column displays the success rate of the particular method (Fpocket or ConCavity) when PRANK is not involved, while the *Rescored* column shows the success rate when PRANK was utilized as a post-processing step. It should be emphasized that since PRANK’s goal is not to discover any new pockets, the maximum achievable success rate is upper bounded by the total coverage of the native prediction method as displayed in the *All* column. In other words, the difference between *Top-n* and *All* represents the possible improvement margin, i.e., the highest nominal improvement in success rate for the *Top-n* cutoff that can be achieved by optimal reordering of the candidate pockets. Thus, the *Improvement* column shows the nominal improvement of PRANK while the *%possible* column shows the percentage of the possible improvement margin. Finally, the last three columns show the statistics related to the PRANK’s underlying Random Forests classifier itself.

**Table 2:** **Rescoring Fpocket and ConCavity predictions with PRANK: cross-validation results on CHEN11 dataset and the results of the final prediction model (trained on CHEN11-Fpocket) for all datasets**

**Dataset**	**Top-n [%]**	**Rescored [%]**	**All [%]**	***Δ***	%possible*	**P**	**R**	**MCC**
Fpocket predictions								
CHEN11 (CV)**	47.9	58.8	71	+10.6	47.1	0.60	0.32	0.41
CHEN11***	47.9	67.9	71	+20	86.4	0.87	1.0	0.98
ASTEX	58	63.6	81.1	+5.6	24.2	0.56	0.41	0.46
DT198	37.5	56.2	80.2	+18.8	43.9	0.31	0.38	0.33
MP210	56.6	67.7	78.8	+11.1	50	0.58	0.42	0.47
B48	74.1	81.5	92.6	+7.4	40	0.58	0.45	0.49
U48	53.7	77.8	88.9	+24.1	68.4	0.55	0.36	0.42
ConCavity predictions								
CHEN11 (CV)**	47.9	50.7	52.3	+2.8	63.3	0.44	0.76	0.40
CHEN11***	47.9	52.3	52.3	+4.4	100	0.80	0.82	0.75
ASTEX	55.2	62.9	65.7	+7.7	73.3	0.60	0.55	0.46
DT198	45.8	61.5	65.6	+15.6	78.9	0.33	0.55	0.34
MP210	57.4	66.1	68.2	+8.7	80.6	0.63	0.53	0.49
B48	66.7	77.8	81.5	+11.1	75	0.61	0.53	0.47
U48	64.8	74.1	77.8	+9.3	71.4	0.58	0.46	0.43

Abbreviations: P precision, R recall, MCC Matthews correlation coefficient.

^*^percentage of improvement that was theoretically possible to obtain by reordering pockets [ *Δ* / (All – Top-n)].

^**^cross-validation results.

^***^results where the test set was de facto the same as the training set for the Random Forest classifier (included here only for completeness).

The results clearly show that the application of PRANK, using the D_CA_ pocket detection criterion with 4 *A* ¨ threshold, considerably outperformed the native ranking methods of Fpocket and ConCavity on all the evaluation datasets. In most of the cases more than 50% of the possible improvement (the *Rescored* column) was achieved. When translated into the absolute numbers, it means that in some cases using PRANK can boost the overall prediction performance of a method by up to 20% (the *Improvement* column) with respect to the absolute achievable maximum.

**Figure 3:**
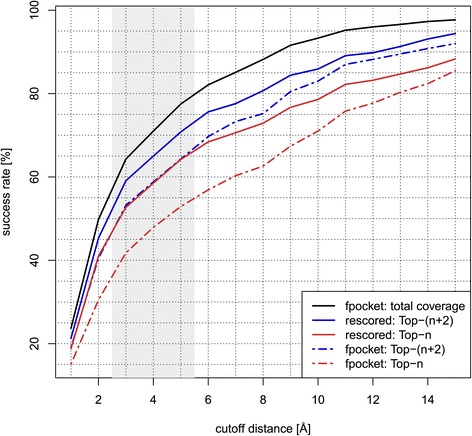
Rescoring Fpocket predictions on CHEN11 dataset. Success rates of Fpocket compared with results rescored by PRANK on CHEN11 dataset considering Top-n, Top-(n+2) and all pockets (total coverage). Identification success is measured by D_CA_ criterion for the range of integer cutoff distances. Displayed results for rescored pockets are averaged from ten independent 5-fold cross-validation runs.

**Figure 4:**
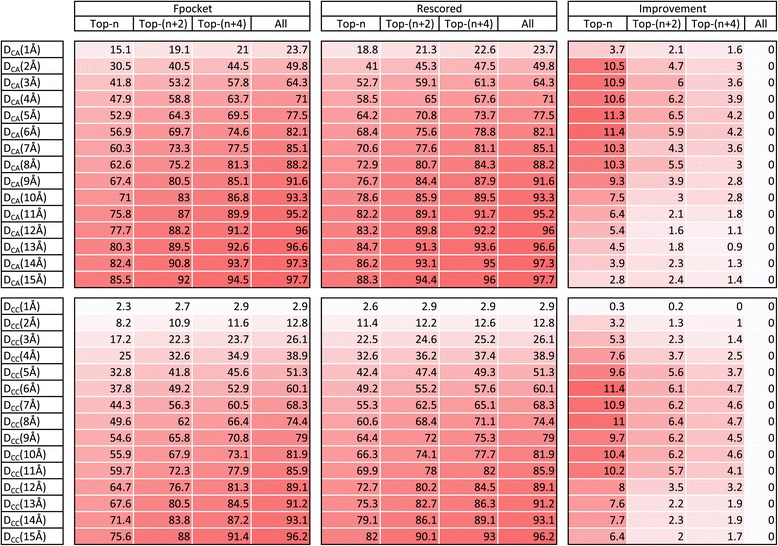
Detailed results. Table and heatmap showing success rates [%] of Fpocket predictions for original and rescored output list of pockets together with the nominal improvements made by PRANK rescoring algorithm on CHEN11 dataset (measured by D_CA_ and D_CC_ criteria for different integer cutoff distances). For the D_CA_ criterion the biggest improvements were achieved around the meaningful 4-6 Å cutoff distances. Displayed results are averaged numbers from ten independent 5-fold cross-validation runs. Four columns in each group show success rates calculated considering progressively more predicted pockets ranked at the top (where *n* is the number of known ligand-binding sites of the protein that includes evaluated binding site). For protein with just one binding site they correspond to Top-1, Top-3 and Top-5 cutoffs that were commonly used to report results in previous ligand-binding site prediction studies.

We also conducted experiments showing how PRANK behaves when the distance threshold in the D_CA_ pocket detection criterion varies. The results carried out on the CHEN11 dataset demonstrate that the improvement of PRANK is basically independent on the utilized threshold (see Figure 3). Finally, to explore the PRANK qualities in greater detail, Figure 4 displays the success rates tracking different distance thresholds and different Top-N cutoffs on the CHEN11-Fpocket dataset.

Furthermore, we compared performance of PRANK against two simpler pocket ranking methods: PLB index, which is based on amino acid composition [29], and simple ordering of pockets by volume that serves as a baseline. PLB index was originally developed to rescore pockets of MOE SiteFinder [15]. We have reimplemented the method and used it to rescore pockets found by Fpocket and ConCavity. The results of the comparison are summarized in Table 3. Using PRANK to rescore Fpocket outperforms both ranking methods on all datasets while for ConCavity predictions PRANK is outperformed only in individual cases by volume ranking on Astex dataset and PLB index on U(B)48 datasets. The improvement by application of PRANK is more significant when rescoring outputs of Fpocket than ConCavity. This can be attributed to the fact that ConCavity predicts, on average, less putative pockets than Fpocket (see Table 1). Having lower margin then allows even a simple method to yield relatively good performance since the possibility of error is lower as well. We can conclude that PRANK is better in prioritizing long lists of pockets that contain many false positives and therefore gives more stable results. All results are summarized in Additional file 2: Tables.

**Table 3:** **PRANK vs. simpler rescoring methods**

**Dataset**	**Top-n [%]**	**All [%]**	**PRANK [%]**	***Δ*** ** PRANK**	**PLB [%]**	***Δ*** ** PLB**	**VOL [%]**	***Δ*** ** VOL**
Fpocket predictions								
CHEN11	47.9	71	58.8**	+10.6	49.8	+1.9	34.5	-13.4
ASTEX	58	81.1	63.6	+5.6	56.6	-1.4	32.2	-25.9
DT198	37.5	80.2	56.2	+18.8	43.2	+5.7	19.3	-18.2
MP210	56.6	78.8	67.7	+11.1	54.5	-2.1	30.6	-26
B48	74.1	92.6	81.5	+7.4	72.2	-1.9	42.6	-31.5
U48	53.7	88.9	77.8	+24.1	66.7	+13	31.5	-22.2
ConCavity predictions								
CHEN11	47.9	52.3	50.7**	+2.8	50.4	+2.5	50.2	+2.3
ASTEX	55.2	65.7	62.9	+7.7	62.9	+7.7	63.6	+8.4
DT198	45.8	65.6	61.5	+15.6	56.8	+10.9	59.4	+13.5
MP210	57.4	68.2	66.1	+8.7	64.9	+7.3	64.6	+6.9
B48	66.7	81.5	77.8	+11.1	79.6	+13	75.9	+9.3
U48	64.8	77.8	74.1	+9.3	75.9	+11.1	70.4	+5.6

PLB - rescoring by the Propensity for Ligand Binding index based on amino acid composition of pockets [29].

VOL - rescoring by approximate volume.

^**^cross-validation results.

The number presented for rescoring methods (columns: PRANK,PLB,VOL) is the success rate considering Top-n predicted pockets measured by D_CA_ criterion with 4 Å threshold.

Although we believe that the overall performance or the PRANK method is good enough, the performance of the underlying prediction model itself can be considered less satisfactory (see the last three columns in Table 2). In few cases the classifier achieved precision of less than 0.5, which means that of all the predicted positives more than a half was predicted incorrectly. Despite of that, reordering pockets according to the new scores led to improvements. This is possible because even predictions deemed as false positives (not within a 2.5 *A* ¨ distance to the ligand) could actually be points from true pockets and contribute to their score. Secondly, because of the particular way we calculate the final pocket score (see Equation 7), even the predictions labeled as negative (having *P*_1_ probability lower than 0.5) contribute to the score to some extent.

### Discussion

Methods based on evolutionary conservation (such as ConCavity and LIGSITE^csc^) are biased towards binding sites with biological ligands (meaning ligands that have their biological function i.e ‘are supposed to bind there’) and therefore can possibly ignore pockets that are not evolutionary conserved but still ligandable with respect to their physico-chemical properties. Those are perhaps the most interesting pockets because among them we can find novel binding sites for which synthetic ligands can be designed. Our method, on the other hand, is based only on local geometric and physico-chemical features of points near protein surface and therefore, we believe, not prone to such bias.

It can be argued that since our model is trained on a particular dataset, it is biased towards binding sites in this dataset. This is inherently a possible issue of all methods that are based on machine learning from examples. However, we believe that by training a classifier to predict ligandability of pocket points (that represent *local* chemical neighborhood rather than the whole pocket) we provided a way for sufficient generalization and therefore ability to correctly predict ligandability of novel sites.

While our rescoring method leads to significant improvements of the final success rates of binding site predictions, performance of the classifier itself is less satisfactory (see Table 2). Here, we will try to outline possible reasons. Several indicators point to the fact that the training data we are dealing with in the classification phase are very noisy.

This can be due to two main reasons: one is related to the feature extraction and the other, more fundamental, has to do with completeness (or rather incompleteness) of the available experimental data.

Regarding the feature extraction, it is possible that (a) our feature set is not comprehensive enough and/or (b) we somehow dilute our feature vectors in the aggregation step mixing positives and negatives. While we cannot rule out the possibility that either could be the case, it is practically impossible to prove such a conclusion.

As for the available experimental data, on the other hand, it is easy to see how their inherent incompleteness could be contributing to the noisiness of our datasets. If we establish some region on protein’s surface as a true ligand-binding site, this—by definition—means that there is an experimentally confirmed 3D structure complex available and thus there exists a ligand which binds at exactly that place. All positives in our datasets are therefore correctly labeled.

What about negatives? Negatives, in our case, are practically represented by everything else or more precisely all other points within the putative pockets. Hence, we can ask the following question: If a point near the protein surface is labeled as negative, does that mean that no ligand could bind at that place (because of its unfavorable physico-chemical properties), or do we simply not have a crystal structure where such event happens? We have no means of giving a definite answer to this question, but we suppose that some pockets are labeled as negatives incorrectly because of the inherent lack of complete experimental data (complete in a sense of confirming/ruling out binding with all possible ligands).

The dataset that was used to train our final classification model (CHEN11) had been constructed in a way that made the presence of false negatives less likely by including all known PDB ligands for the proteins present in the dataset. It is possible that it would prove better to work with much more narrowly defined negatives, that is, to take our negatives only from the putative pockets for which no ligand has been found despite a deliberate effort. However, this approach would have its own problems since examples of such cases are quite rare [30,52] and although they exist, they do not cover all structural diversity of whole PDB the way CHEN11 dataset does. Moreover, there are known cases when a ligand has been found for pockets that were previously deemed unligandable [53]. Another source of more reliable negatives could be proteins deemed unligandable by physical fragment screens [54]. Nonetheless, as it could be quite interesting to see the effect it would have on the performance of our method, we shall leave it for the future research.

## Conclusion

We introduced PRANK, a novel method to be used as a post processing step to any pocket identification method providing a rescoring mechanism to prioritize the predicted putative pockets. Since pocket prediction tools output many false positive results, a subsequent prioritization step can greatly boost the performance of such tools. PRANK is based on machine-learning providing the ability to predict ligandability of specific pocket points. The predictions are combined into a score for a given putative pocket which is then used in the re-ranking phase. As demonstrated on multiple datasets using the examples of Fpocket and ConCavity, the method consistently increases the performance of the pocket detection methods by correct prioritization of the putative sites. PRANK is distributed as a freely available tool currently capable to work with the outputs of Fpocket and ConCavity, but it can be easily adapted to process an output from basically any pocket prediction tool. We believe that we have addressed a previously neglected problem of pocket scoring and thus the introduced method and the accompanying software present a valuable addition to the array of publicly available cheminformatics tools. PRANK is freely available at http://siret.ms.mff.cuni.cz/prank.

## Endnote

^a^ Although version 2.0 of Fpocket in its beta was available, we decided to use the version 1.0 since it consistently yielded better results.

## Additional files

Additional file 1
**Listings.** Document that contains supplementary listings: (1) the complete list of properties of feature vectors used to represent inner points and (2) the lists of proteins by dataset for which ConCavity was run with the conservation mode switched off.

Additional file 2
**Tables.** Excel file that contains data used to produce tables and figures in this article.
