# Supplementary material for: Improving protein-ligand binding site prediction accuracy by classification of inner pocket points using local features
Source: J Cheminform. 2015 Apr 1;7:12. doi: 10.1186/s13321-015-0059-5 (PMC4414931; doi:10.1186/s13321-015-0059-5)
Supplement: Additional file 1 — Listings. Document that contains supplementary listings: (1) the complete list of properties of feature vectors used to represent inner points and (2) the lists of proteins by dataset for which ConCavity was run with the conservation mode switched off. [file 13321_2015_59_MOESM1_ESM.pdf]

Additional Listings File

Supplementary information to the article

Improving ligand-binding site prediction accuracy by classification of inner pocket points using local features

Radoslav Kriváк and David Hoksza

1. Complete list of features

Complete list of properties of feature vectors used to represent inner pocket points.

Feature name	description
`hydrophobic`	binary attribute, 1 for hydrophobic residues
`hydrophilic`	binary attribute, 1 for hydrophilic residues
`hydrophatyIndex`	side-chain hydrophaty index with values in range $\langle -4.5, 4.5 \rangle$ [1]
`aliphatic`	binary attribute, 1 for aliphatic residues
`aromatic`	binary attribute, 1 for aromatic residues
`sulfur`	binary attribute, 1 for residues containing sulfur
`hydroxyl`	binary attribute, 1 for hydroxyl group containing residues
`basic`	binary attribute, 1 for basic residues
`acidic`	binary attribute, 1 for acidic residues
`amide`	binary attribute, 1 for amide group containing residues
`posCharge`	binary attribute, 1 for positively charged residues
`negCharge`	binary attribute, 1 for negatively charged residues
`hBondDonor`	binary attribute, 1 for H-bond donor containing residues
`hBondAcceptor`	binary attribute, 1 for H-bond acceptor containing residues
`hBondDonorAcceptor`	binary attribute, 1 for residues that have H-bond donor AND acceptor
`polar`	binary attribute, 1 for polar residues
`ionizable`	binary attribute, 1 for ionizable residues
`atoms`	absolute number of protein exposed atoms (within 8 Å radius of the point)
`atomDensity`	number of protein exposed atoms weighted by distance
`atomC`	number of carbon atoms
`atomO`	number of oxygen atoms
`atomN`	number of nitrogen atoms
`hDonorAtoms`	number of H-bond donor atoms

hAcceptorAtoms	number of H-bond acceptor atoms
vsAromatic	VolSite atomic level features [2]
vsCation	~
vsAnion	~
vsHydrophobic	~
vsAcceptor	~
vsDonor	~
ap5sasaValids	Ligand binding propensity for biologically valid ligands [3]
ap5sasaInvalids	Ligand binding propensity for biologically invalid ligands [3]
protrusion	Protein surface protrusion inspired by [4] calculated simply as number of all protein atoms (not just exposed) within 10 Å radius of the point
bfactor	B-factor number of the atom from pdb file

References:

1. Kyte, J., Doolittle, R.F.: A simple method for displaying the hydropathic character of a protein. Journal of Molecular Biology 157(1), 105–132 (1982).
2. Desaphy, J., Azdimousa, K., Kellenberger, E., Rognan, D.: Comparison and druggability prediction of protein-ligand binding sites from pharmacophore-annotated cavity shapes. Journal of chemical information and modeling 52(8), 2287–2299 (2012).
3. Khazanov, N.A., Carlson, H.A.: Exploring the composition of protein-ligand binding sites on a large scale. PLoS computational biology 9(11), 1003321 (2013).
4. Alessandro Pintar, Oliviero Carugo, and Sándor Pongor: CX, an algorithm that identifies protruding atoms in proteins. Bioinformatics (2002) 18 (7): 980-984

2. ConCavity no-conservation proteins

Lists of proteins (by dataset) for which ConCavity was run with the conservation mode switched off. Those are the proteins for which pre-computed sequence conservation files were not available or ConCavity failed to produce any results in conservation mode even if they were.

CHEN11 - 16/251	a.003.001.004_1m6zb.pdb<br>a.138.001.003_1qdbb.pdb<br>b.042.002.001_2zqnb.pdb<br>b.085.007.002_2g46a.pdb<br>b.089.001.001_1iiya.pdb<br>c.001.013.001_1p7tb.pdb<br>c.002.001.003_2g82c.pdb<br>c.025.001.004_1ja1b.pdb<br>c.062.001.001_3bqmb.pdb<br>c.065.001.001_2blnb.pdb<br>c.087.001.001_1m5rb.pdb

	c.087.001.010_2c1xa.pdb<br>d.001.001.004_1bvic.pdb<br>d.019.001.001_2akrc.pdb<br>d.110.006.001_1p0zg.pdb<br>e.003.001.001_2hdub.pdb
ASTEX - 2/85	1hnn.pdb<br>1oyt.pdb
UB48 - 5/96	1dwd.pdb<br>1hxf.pdb<br>1ida.pdb<br>1pso.pdb<br>3gch.pdb
DT198 - 19/198	1cea_A.pdb<br>1fj8_A.pdb<br>1lxf_C.pdb<br>1pk2_A.pdb<br>1q8y_B.pdb<br>1y4l_B.pdb<br>2cft_A.pdb<br>2xh1_A.pdb<br>2xhd_A.pdb<br>2zt7_A.pdb<br>3d90_A.pdb<br>3gmz_A.pdb<br>3h6t_A.pdb<br>3ii0_A.pdb<br>3inj_A.pdb<br>3iyt_A.pdb<br>3k4v_A.pdb<br>3kvv_A.pdb<br>3l6b_A.pdb
MP210 - 4/210	1ac0.pdb<br>1b6n.pdb<br>2er0.pdb<br>3gch.pdb
